# Serum Angiopoietin‐Like Protein 4 as a Biomarker for Acute Ischemic Stroke Severity and Dynamic Changes

**DOI:** 10.1002/brb3.70337

**Published:** 2025-02-19

**Authors:** Fang Guo, Chong Zheng, Tao Yang, Fang‐Wei Hu, Yan‐Gui Chen, Chia‐Wei Liou, Bin Cai

**Affiliations:** ^1^ Department of Neurology and Institute of Neurology, First Affiliated Hospital, Institute of Neuroscience, Fujian Key Laboratory of Molecular Neurology Fujian Medical University Fuzhou China; ^2^ Department of Neurology Longyan First Affiliated Hospital of Fujian Medical University Longyan China; ^3^ Department of Neurology, National Regional Medical Center, Binhai Campus of the First Affiliated Hospital Fujian Medical University Fuzhou China; ^4^ Department of Neurology Kaohsiung Chang Gung Memorial Hospital and Chang Gung University College of Medicine Kaohsiung Taiwan

**Keywords:** angiopoietin‐like protein 4, acute ischemic stroke, NIHSS

## Abstract

**Background:**

Angiopoietin‐like protein 4 (ANGPTL4) is critical for vascular integrity and reducing inflammation in ischemic and hypoxic brain injuries. However, limited studies have evaluated ANGPTL4's role in acute ischemic stroke (AIS) assessment, and its expression patterns across AIS phases remain unclear.

**Methods:**

The severity of AIS at admission was assessed using the National Institutes of Health Stroke Scale (NIHSS). The association between serum ANGPTL4 level and the occurrence of AIS was examined using logistic regression analysis. The diagnostic accuracy of serum ANGPTL4 level for AIS severity was assessed using receiver operating characteristic curves.

**Results:**

This study included 389 AIS patients and 133 healthy individuals. There was a notable increase in the occurrence of AIS associated with rising serum ANGPTL4 levels (odds ratio [OR] 1.03, 95% confidence interval [CI]: 1.02–1.06; *p* < 0.001). A higher serum level of ANGPTL4 was also found to be associated with severe AIS, as indicated by an AUC of 0.848. Additionally, we observed significant dynamic changes in ANGPTL4 levels, with a marked decrease at 1 week or 2 weeks after admission compared with the acute phase (the day after admission; both *p* < 0.001).

**Conclusions:**

Our findings suggest a robust association between elevated serum ANGPTL4 levels and the presence and severity of AIS. Importantly, this study is distinguished by its novel focus on the temporal dynamics of ANGPTL4 levels, which underscores its potential as a biomarker for AIS monitoring and provides new insights into AIS pathophysiology.

## Graphical abstract

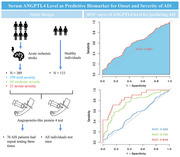

## 1 Introduction

Stroke is globally recognized as the second leading cause of death and a major contributor to disability (Campbell et al. 2019; Gorelick 2019). Ischemic stroke, representing 71% of all stroke cases worldwide, mainly results from arterial blockages (Feigin et al. 2018). In acute ischemic stroke (AIS), the brain area affected, known as the ischemic core, rapidly expands (Campbell et al. 2019). The penumbra, the surrounding brain tissue, is crucial in clinical symptoms and can recover if blood flow is quickly restored (Campbell et al. 2019). Therefore, immediate reperfusion through intravenous thrombolysis and endovascular therapy is essential in AIS management (Powers et al. 2019; Shobha, Buchan, and Hill 2011; Nogueira et al. 2018). Prompt and accurate diagnosis of AIS and its severity is key to achieving optimal patient outcomes (Shobha, Buchan, and Hill 2011; Nogueira et al. 2018).

However, computed tomography (CT) and magnetic resonance imaging (MRI), commonly used for AIS diagnosis, are not always accessible due to high costs and the need for specialized expertise (Hasan et al. 2018; Musuka et al. 2015). The National Institutes of Health Stroke Scale (NIHSS) is widely used to assess stroke severity, but its dependence on skilled neurologists limits its use in economically disadvantaged areas and smaller medical facilities. Delays in stroke evaluation and treatment often lead to poor patient outcomes (Patil et al. 2022). This highlights the urgent need for a rapid, affordable, and accessible method to diagnose AIS and evaluate its severity early on.

Serological markers are increasingly recognized as promising indicators for AIS due to their noninvasive nature and diagnostic accuracy (Alhazzani et al. 2021). Among these markers, angiopoietin‐like protein 4 (ANGPTL4), a secreted proangiogenic protein, has attracted attention for its role in ischemic conditions (Santulli 2014; Le Jan et al. 2003). Studies have demonstrated elevated ANGPTL4 expression in both mouse and human brain tissues under hypoxic and ischemic conditions (Wiesner et al. 2006; Bouleti et al. 2013). In AIS patients, higher serum levels of ANGPTL4 have been observed, with significant correlations to NIHSS scores and lesion size, suggesting its clinical relevance (He et al. 2016). Mechanistically, ANGPTL4 is hypothesized to play a protective role in ischemic stroke by maintaining vascular integrity, modulating postischemic inflammation, and mitigating adverse outcomes such as edema, neuron loss, and infarct size, as shown in animal models (Bouleti et al. 2013; Xu et al. 2015; Qiu et al. 2021; Spescha et al. 2013). These findings highlight ANGPTL4's potential not only as a biomarker for diagnosing AIS and assessing its severity but also as a therapeutic target for improving vascular and neural outcomes.

However, there is a lack of studies confirming ANGPTL4's effectiveness in assessing AIS, and its specific expression patterns during different AIS phases are not well understood (He et al. 2016). Our study aims to evaluate the diagnostic potential of ANGPTL4 for AIS and explore the temporal dynamics of ANGPTL4 in relation to AIS.

## 2 Material and Methods

### 2.1 Data Sources and Participants

From August 2017 to May 2021, a clinical observational study was conducted, recruiting first‐time AIS patients from the Neurology Departments of the First Affiliated Hospital of Fujian Medical University and Longyan First Affiliated Hospital of Fujian Medical University in China. The study was approved by the ethics committees of both hospitals. Approval from the Branch for Medical Research and Clinical Technology Application, Ethics Committee of the First Affiliated Hospital of Fujian Medical University (approval number [2016]094) was granted on June 6, 2016. Similarly, approval from the Ethics Committee of the Longyan First Affiliated Hospital of Fujian Medical University (approval number [2017]034) was obtained on June 16, 2017. The study adhered to the principles of the Declaration of Helsinki, and informed consent was obtained from all participants or their immediate family members prior to inclusion. Patients admitted within 24 h of symptom onset and had not underwent endovascular or intravenous thrombolysis treatments were selected and diagnosed according to the World Health Organization criteria (1988). To confirm AIS and rule out other conditions like hemorrhagic stroke, diagnostic imaging (CT or MRI) was performed within five days of symptom onset. Based on stroke severity, AIS patients were categorized into mild, moderate, and severe groups.

The clinical observational study also involved healthy individuals who were hospitalized during the same period and finally diagnosed as having no cerebrovascular diseases. Exclusion criteria included a history of myocardial infarction, serious infections, autoimmune or blood diseases, thyroid disorders, chronic obstructive pulmonary disease, traumatic brain injury, cerebral hemorrhage, intracranial mass, brain surgery, aneurysm, severe organ failures, and known malignant tumors.

**FIGURE 1:**
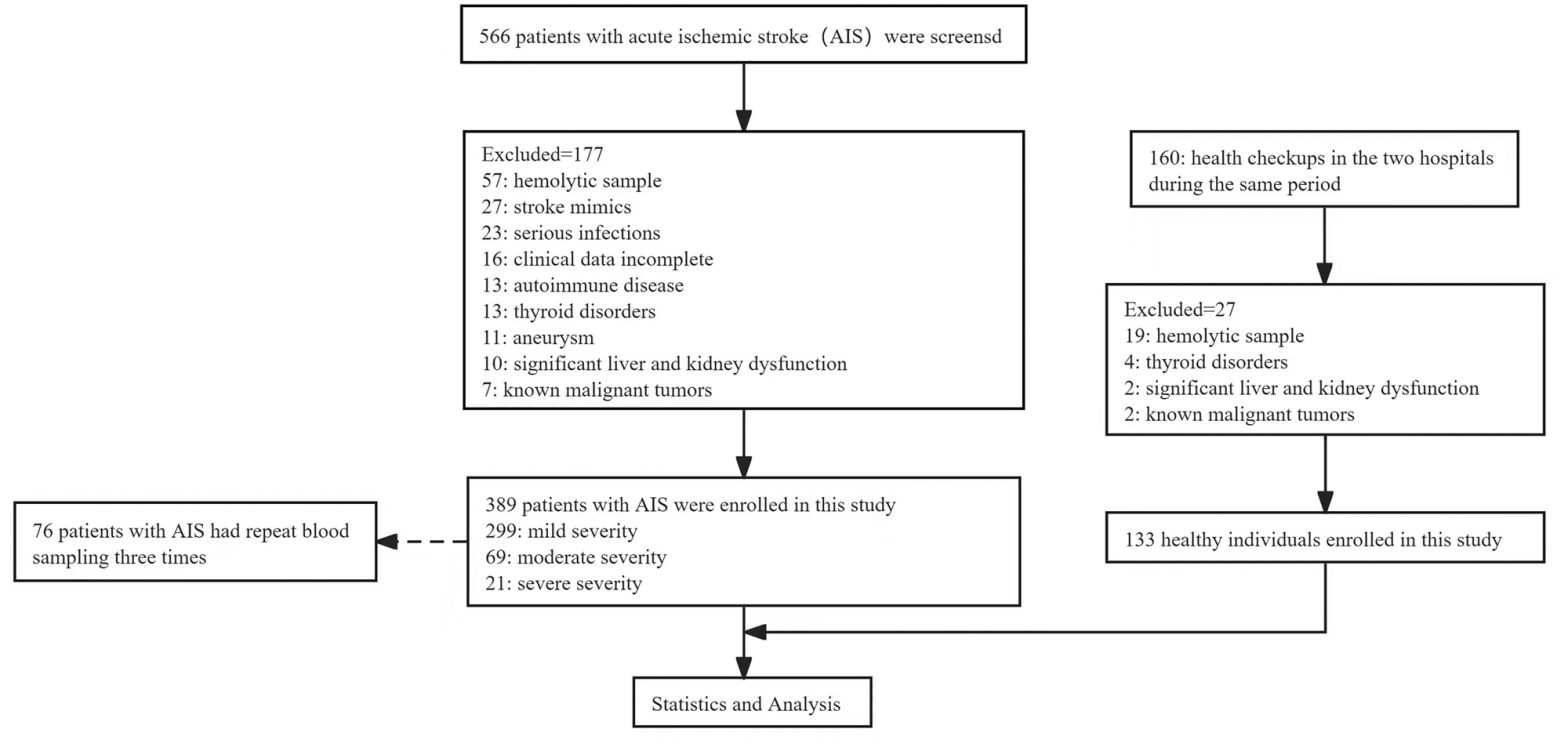
A study flow diagram.

In total, 389 patients aged 27 or older were enrolled, divided into 299 with mild AIS, 69 with moderate AIS, and 21 with severe AIS. Additionally, 133 healthy individuals served as controls (Figure 1).

### 2.2 Data Collection

Baseline data were collected for each participant or acquired from their family members. This encompassed demographic information such as age and gender, as well as lifestyle factors, including smoking and drinking habits. The medical histories of participants, highlighting conditions like hypertension, diabetes mellitus, coronary artery disease, and atrial fibrillation, were also recorded. All participants underwent comprehensive laboratory tests at the involved hospital. These tests measured various blood components, including low‐density lipoprotein cholesterol (LDL‐C), high‐density lipoprotein cholesterol (HDL‐C), total cholesterol (T‐CHOL), triglycerides (TG), homocysteine (HCY), and uric acid (UA). Additionally, each participant received a cervical vascular color doppler examination to assess the carotid intima‐media thickness (IMT) and the presence of carotid plaque, both key indicators of cardiovascular health. Type I plaques are hypoechoic lesions with a thin echogenic fiber cap; type II plaques are predominantly hypoechoic, but the echogenic region accounts for less than 25% of the plaque; type I and type II plaques are graded as unstable carotid plaques; type III plaques was a predominantly hyperechoic lesion with hypoechoic areas representing less than 25% of the plaque; type IV plaques was a homogenously hyperechoic plaque; types III and IV were graded as stable plaques (Saba et al. 2014; Ammirati et al. 2015). Furthermore, two specific stroke‐related assessments were conducted by trained stroke physicians at the time of patient admission. These included the NIHSS score (Powers et al. 2018), which quantifies stroke severity, and the Essen stroke risk score (ESRS) (Weimar et al. 2008), which helps determine the risk of future strokes in patients.

### 2.3 The Measurement of Serum ANGPTL4 Level

Fasting venous blood samples were taken from participants within 24 h of their enrollment, following a minimum 8 h fasting period, to measure serum ANGPTL4 level. These samples were then centrifuged at 3800 rpm for 10 min. In instances where hemolysis was not present, the top serum layer was extracted and stored at −80°C for subsequent analysis. For some patients, this procedure was repeated at three different intervals: the next day, 1 week (7 ± 1 days), and 2 weeks (14 ± 1 days) after hospital admission.

To determine the serum ANGPTL4 level, we used a commercial ELISA kit (Raybiotech, Norcross, GA, USA), adhering to the manufacturer's guidelines. This kit could detect ANGPTL4 levels as low as 20 pg/mL. The assay's precision was ensured with an intra‐assay coefficient of variation (CV%) of less than 10% and an inter‐assay CV% of less than 12%. All samples were tested in duplicate, and the results were reported in picograms of ANGPTL4 per milliliter of serum. To maintain objectivity, the laboratory technicians conducting these measurements were not informed about the clinical characteristics or outcomes of the study participants.

### 2.4 The Assessment of AIS Severity

AIS severity was classified into three categories based on the NIHSS score: mild (NIHSS score less than 8), moderate (NIHSS score between 8 and 15), and severe (NIHSS score greater than 15; (Kirchhof P et al. 2016)).

### 2.5 Statistical Analysis

In this study, the Shapiro‐Wilk test was used to assess the normality of the data. Continuous variables that followed a normal distribution were presented as means (±standard deviation [SD]), while those not normally distributed were shown as medians (with 25% and 75% quartiles). Categorical variables were expressed as proportions. To compare baseline characteristics between groups, the Student's *t*‐test or the Wilcoxon rank‐sum test was used for continuous variables, and the chi‐square test was used for categorical variables.

The association between serum ANGPTL4 level and the occurrence of AIS was examined using logistic regression analysis. This analysis was conducted in three stages: unadjusted, adjusted for age and gender, and then adjusted for all significant covariates identified in the univariable analysis. Adjusted odds ratios (OR) with 95% confidence intervals (CI) were presented to describe these relationships. To evaluate serum ANGPTL4 levels across different AIS severity categories, the Kruskal–Wallis test was applied. The diagnostic accuracy of serum ANGPTL4 level for various AIS severities was assessed using receiver operating characteristic (ROC) curves, and the area under the curve (AUC) was calculated. An AUC above 0.80 indicated high diagnostic efficacy, 0.5–0.8 suggested moderate efficacy and an AUC of 0.5 or less was considered insignificant. The Youden index was used to determine the optimal cut‐off value. All of the above analyses of ANGPTL4 were performed using values from the day after hospital admission. Furthermore, the Friedman test was employed to compare serum ANGPTL4 levels at different time points during AIS, with the Bonferroni correction being applied for multiple comparisons.

A two‐tailed *p*‐value of less than 0.05 was considered statistically significant. All statistical analyses were performed using the R software (version 4.2.1; R Foundation for Statistical Computing, Vienna, Austria).

## 3 Result

### 3.1 Clinical Characteristics of Study Participants

In this study, 522 participants were enrolled. The baseline demographic and clinical characteristics are shown in Table 1. Compared with healthy individuals, AIS patients displayed several distinct traits. They were more likely to be older (*p* = 0.030), male (*p* < 0.001), and smokers (*p* = 0.014). They also had higher incidences of hypertension (*p* < 0.001), diabetes mellitus (*p* = 0.004), and atrial fibrillation (*p* < 0.001). Clinically, AIS patients typically had lower HDL‐C levels (*p* < 0.001), higher carotid IMT (*p* < 0.001), unstable carotid plaque (*p* < 0.001), and elevated ESRS (*p* < 0.001). Significantly higher serum ANGPTL4 level was observed in the AIS group compared with the healthy control group (*p* = 0.003), with level in the AIS group at 1113.30 pg/mL (interquartile range [IQR]: 673.42–1967.50 pg/mL), and in the healthy control group at 876.18 pg/mL (IQR: 571.68–1523.05 pg/mL).

**TABLE 1:** Baseline demographic and clinical characteristics of study participants.

Characteristic	Overall (*N* = 522)	AIS group (*N* = 389)	Healthy control group (*N* = 133)	*p*‐value
Age, years	64.3 ± 11.8	65.0 ± 11.7	62.4 ± 11.6	0.030
Female, *n* (%)	200 (38.3)	129 (33.2)	71 (53.4)	<0.001
Smoking, *n* (%)	210 (40.2)	169 (43.4)	41 (30.8)	0.014
Drinking, *n* (%)	89 (17.0)	69 (17.7)	20 (15.0)	0.561
LDL‐C, mmol/L	3.17 ± 1.06	3.21 ± 1.10	3.05±0.94	0.128
HDL‐C, mmol/L	1.28 ± 0.39	1.24±0.38	1.39 ± 0.41	<0.001
UA, µmol/L	344.65 ± 98.27	344.37 ± 100.66	345.45 ± 91.30	0.913
T‐CHOL, mmol/L	4.79 ± 1.16	4.82±1.21	4.69 ± 1.02	0.274
TG, mmol/L	1.31 [0.92, 1.80]	1.36 [0.96, 1.85]	1.16 [0.86, 1.59]	0.064
HCY, µmol/L	11.51 [8.77, 15.77]	11.67 [8.64, 16.10]	10.99 [8.90, 14.90]	0.462
ANGPTL4, pg/mL	1062.45 [649.87, 1791.22]	1113.30 [673.42, 1967.50]	876.18 [571.68, 1523.05]	0.003
Carotid IMT, mm	1.00 ± 0.19	1.02 ± 0.18	0.92 ± 0.20	<0.001
Nature of carotid plaque, *n* (%)				
No plaque	133 (25.5)	75 (19.3)	58 (43.6)	<0.001
Unstable plaque	295 (56.5)	241 (62.0)	54 (40.6)	
Stable plaque	94 (18.0)	73 (18.8)	21 (15.8)	
ESRS	2.00 [1.00, 3.00]	3.00 [2.00, 3.00]	2.00 [1.00, 3.00]	< 0.001
Hypertension, *n* (%)	379 (72.6)	310 (79.7)	69 (51.9)	< 0.001
Diabetes mellitus, *n* (%)	151 (28.9)	126 (32.4)	25 (18.8)	0.004
Coronary artery disease, *n* (%)	36 (6.9)	23 (5.9)	13 (9.8)	0.187
Atrial fibrillation, *n* (%)	51 (9.8)	49 (12.6)	2 (1.5)	< 0.001

Abbreviations: AIS, acute ischemic stroke; ANGPTL4, angiopoietin‐like protein 4; ESRS, Essen stroke risk score.; HCY, homocysteine; HDL‐C, high‐density lipoprotein cholesterol; IMT, intima‐media thickness; LDL‐C, low‐density lipoprotein cholesterol; T‐CHOL, total cholesterol; TG, triglycerides; UA, uric acid.

### 3.2 The Diagnostic Value of Serum ANGPTL4 Level for AIS

Logistic regression analysis, as shown in Table 2, indicated a significant association between serum ANGPTL4 level and the likelihood of AIS. In the univariate model, an increase in serum ANGPTL4 level was linked to a higher AIS likelihood (OR: 1.03, 95% CI: 1.02–1.06; *p* < 0.001). This association persisted in age‐ and sex‐adjusted models (OR: 1.03; 95%CI: 1.02–1.05; *p* = 0.002) and even after adjusting for all significant covariates from the univariable analysis, where each 100 pg/mL increase in serum ANGPTL4 level correlated with a four percent rise in AIS likelihood (OR: 1.04, 95% CI: 1.01–1.06; *p* < 0.001). Quartile analyses revealed linear increases of AIS likelihood with an increase of serum ANGPTL4 level (*p* for trend <0.05 for all models).

**TABLE 2:** The prevalence ratios and 95% confidence intervals for serum ANGPTL4 levels and AIS.

	Model 1			Model 2			Model 3		
ANGPTL4	OR (95%CI)	*p*‐value	*p*‐value for rend	OR (95%CI)	*p*‐value	*p*‐value for trend	OR (95%CI)	*p*‐value	*p*‐value for trend
Continuous									
ANGPTL4 (per 100 pg/mL)	1.03 (1.02–1.06)	< 0.001	—	1.03 (1.02–1.05)	0.002	—	1.04 (1.01–1.06)	< 0.001	—
Categories									
Group 1	1[Ref]	NA	0.004	1[Ref]	NA	0.015	1[Ref]	NA	0.010
Group 2	0.92 (0.54–1.56)	0.757		0.84 (0.49–1.44)	0.522		0.92 (0.50–1.68)	0.780	
Group 3	1.25 (0.72–2.16)	0.428		1.02 (0.58–1.79)	0.947		1.02 (0.54–1.93)	0.960	
Group 4	2.35 (1.30–4.38)	0.006		2.17 (1.18–4.09)	0.014		2.63 (1.32–5.41)	0.007	

*Note*: Model 1: Not adjusted. Model 2: Adjusted by age and gender. Model 3: Adjusted by age, gender, smoking, hypertension, diabetes mellitus, atrial fibrillation, high‐density lipoprotein cholesterol, carotid intima‐media thickness, carotid plague degrees, and Essen stroke risk score.

Abbreviations: AIS, acute ischemic stroke; ANGPTL4, angiopoietin‐like protein 4; CI: confidence intervals.; OR, odds ratios.

Significant differences in serum ANGPTL4 levels among AIS severities were noted. Both moderate and severe AIS groups had significantly higher serum ANGPTL4 levels than the mild AIS group (*p* < 0.001) and the healthy control group (*p* < 0.001). However, no significant difference was found between moderate and severe AIS groups or between mild AIS and healthy control groups. These findings are detailed in Table 3.

**TABLE 3:** The difference in serum ANGPTL4 levels among individuals with different severities of AIS.

	*N*	ANGPTL4 (pg/mL, P50 (P25, P75)
Healthy control group	133	876.18(571.68, 1523.05)
Mild AIS group	299	992.08(601.12, 1513.55)
Moderate AIS group	69	1883.95(1113.30, 2863.30)^a^, ^b^
Severe AIS group	21	2465.55(1501.20, 5648.10)^a^, ^b^
H		36.073
*p*‐value		< 0.001

*Note*: To analyze serum ANGPTL4 levels among different AIS severities, the Kruskal–Wallis test was applied (healthy control group vs. mild AIS group *p* = 1.000, healthy control group vs. moderate AIS group and severe AIS group *p *< 0.001, mild AIS group vs. moderate AIS group and severe AIS group *p* < 0.001, moderate AIS group vs. severe AIS group p = 0.771).

Abbreviations: AIS, acute ischemic stroke; ANGPTL4, angiopoietin‐like protein 4.

^a^ Sign compared with the healthy control group, *p *< 0.001.

^b^ Sign compared with the mild AIS group, *p *< 0.001.

**FIGURE 2:**
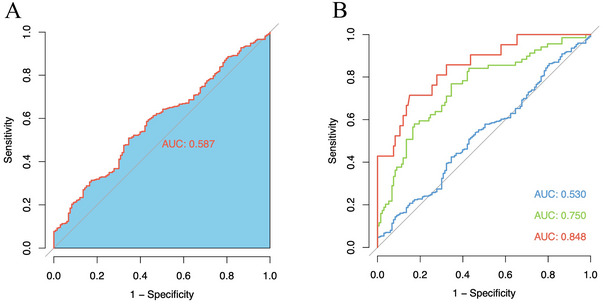
Receiver operating characteristic (ROC) curve analyses. The *y*‐axis meant the true positive rate of the risk prediction. The *x*‐axis meant the false‐positive rate of the risk prediction. A, The ROC curve of the serum angiopoietin‐like protein 4 (ANGPTL4) level for predicting the occurrence of acute ischemic stroke (AIS); area under the curve (AUC) for AIS was 0.587. B, The ROC curves of the serum ANGPTL4 level for predicting the occurrence of different severity AIS. AUC for mild AIS (BLUE symbols and connecting lines) was 0.530. AUC for moderate AIS (GREEN symbols and connecting lines) was 0.750. AUC for severe AIS (RED symbols and connecting lines) was 0.838.

Figure 2 shows that serum ANGPTL4 level effectively distinguished severe AIS from other severities. The optimal cutoff for diagnosing severe AIS was established at 1801 pg/mL, indicating high diagnostic performance with an AUC of 0.848.

### 3.3 Temporal Dynamics of Serum ANGPTL4 Level in AIS

Table 4 tracks the temporal dynamics of serum ANGPTL4 levels in AIS patients. A significant decrease in level was observed from the acute phase (the day after admission) to the 1 week and 2 weeks postadmission (*p* < 0.001). No significant difference was noted between the serum ANGPTL4 level at 1 week and 2 weeks after admission (*p* = 1.000).

**TABLE 4:** Temporal dynamics of serum ANGPTL4 levels in the AIS group.

Time	ANGPTL4(pg/mL, P50(P25, P75))
The next day after admission	1433.93(921.24, 2261.16)
1 week after admission	784.79(511.77, 1194.20)^a^
2 weeks after admission	817.87(513.33, 1251.16)^a^
χ^2^	51.342
*p*‐value	< 0.001

*Note*: Due to various reasons, such as patient nonconsent or sample hemolysis, serum ANGPTL4 levels were successfully measured thrice in 76 patients with AIS.

Abbreviations: AIS, acute ischemic stroke; ANGPTL4, angiopoietin‐like protein 4.

^a^ Sign compared with the day after admission, *p *< 0.001.

## 4 Discussion

The study's primary results show a strong association between elevated serum ANGPTL4 levels and the occurrence of AIS. Notably, this marker was associated with AIS, and its elevated levels were linked to increased AIS severity. This correlation persisted even after adjusting for various confounders. A significant observation was the dynamic change in serum ANGPTL4 levels, with an increase during the acute phase, followed by a marked decrease in the subacute phase one week after AIS onset. This acute‐to‐subacute phase shift in ANGPTL4 expression suggests its potential utility as a biomarker to monitor AIS progression and to assist in early clinical decision‐making.

A disruption of the blood–brain barrier (BBB) is a critical event in AIS, leading to neurological dysfunctions such as vasogenic edema, hemorrhagic transformation, and neuroinflammation (Yang et al. 2019). ANGPTL4 plays a protective role in this context, inhibiting VEGF‐induced BBB leakage by limiting Src kinase activity and activating the PI3K–Akt pathway (Bouleti et al. 2013; Qiu et al. 2018; Gomez Perdiguero et al. 2016). Animal studies have suggested that ANGPTL4 expression may be upregulated as a protective response in AIS (Bouleti et al. 2013; Qiu et al. 2021; Zhang et al. 2017). Our analysis revealed elevated serum ANGPTL4 levels in AIS patients, showing a significant positive association with the occurrence of AIS. Notably, these levels correlated positively with clinical deficits at admission, as measured by the NIHSS, aligning with findings from a previous ischemic stroke study (He et al. 2016). Despite various AIS causes, our study found no difference in the disruption of vascular integrity and inflammatory response mechanisms across different stroke subtypes. In line with the findings of Jin X et al., which indicate that BBB injury develops more rapidly in brain regions with moderate to severe ischemia (Jin X et al. 2012), our study corroborates this perspective. We observed that, compared with other groups, serum ANGPTL4 level was significantly elevated in the moderate and severe AIS groups. This increase in serum ANGPTL4 level in patients with more severe ischemia reinforces the potential role of ANGPTL4 not only as a marker for AIS presence but also as an indicator of its severity. These consistent findings across different studies highlight the importance of ANGPTL4 in the pathophysiology of AIS, particularly in relation to BBB integrity and the severity of ischemic damage.

Our study uniquely highlights the temporal dynamics of serum ANGPTL4 levels following the onset of AIS. We observed an increase in these levels during the acute phase of AIS, followed by a decrease in the subacute phase. This finding is particularly significant, as it aligns with the critical window for biomarker‐based decision‐making in AIS, often considered essential within the first seven days poststroke (Uphaus et al. 2019). The observed pattern in the ANGPTL4 level mirrors the postischemic changes in BBB dysfunction (Bernardo‐Castro et al. 2020). In AIS, peaks in BBB permeability typically occur during the hyperacute stage (less than 6 h) and the acute stage (6–72 h; Strbian et al. 2008; Pillai et al. 2009; Lin et al. 2008, Durukan et al. 2009). This suggests that the protective effects of ANGPTL4 on the BBB might be a key mechanism driving its elevated expression during the acute phase of AIS. However, further research is necessary to confirm this hypothesis and to fully understand the role of ANGPTL4 in the pathophysiology of AIS. Dynamic detection of ANGPT4 in the clinic can assist to some extent in recognizing the severity and in‐hospital changes in the condition of patients with stroke, especially those who are not amenable to repeated in‐hospital cranial CT and MRI evaluations, in order to advise clinicians in their decision‐making. Moreover, our study's revelation of the temporal changes in ANGPTL4 level adds a new dimension to the understanding of AIS progression and offers a potential avenue for timely and targeted therapeutic interventions.

Our study highlights the potential of ANGPTL4 as a key factor in AIS and outlines practical directions for future research. Investigating the mechanisms by which ANGPTL4 protects BBB integrity during ischemic injury could be achieved through advanced imaging and molecular techniques, offering insights into its role in regulating BBB permeability. Additionally, the observed temporal changes in ANGPTL4 levels suggest its potential as a biomarker for optimizing treatment timing and selection, such as thrombolysis or thrombectomy. Longitudinal studies with larger and more diverse cohorts are needed to validate these findings and explore variations across populations. These focused investigations could accelerate the clinical translation of ANGPTL4 in AIS management.

Our study indeed offers important insights but also has several acknowledged limitations. First and foremost, the study was conducted with a relatively small sample size, and this is particularly noticeable in the severe AIS group. Such a limitation could potentially impact the robustness and the broader applicability of our findings. Second, the scope of our analysis was confined to Chinese individuals. As a result, the conclusions drawn from our study might not be fully representative or applicable to other racial or ethnic groups. Third, an important limitation of our study is that serum ANGPTL4 level was not measured in stroke patients before the onset of AIS. This means that we lack data on the baseline serum ANGPTL4 level in patients prior to the occurrence of AIS. This gap in data prevents a comprehensive understanding of the changes in ANGPTL4 level and their significance in the development of AIS. Lastly, considering the role of ANGPTL4 in maintaining the integrity of the BBB, our study's conclusions would have been more compelling if they included the observation of dynamic changes in the BBB.

## 5 Conclusion

Our study demonstrated that ANGPTL4 plays a key role in the development of AIS. These findings suggest the potential of ANGPTL4 to be employed in clinical practice as a screening biomarker. This could significantly aid in the selection of patients for timely and appropriate treatment interventions, enhancing the overall management of AIS. Furthermore, our findings open new research avenues, including the exploration of ANGPTL4 as a therapeutic target to protect BBB integrity and reduce ischemic damage. Future studies should focus on validating the clinical utility of ANGPTL4 in diverse populations and investigating its role in guiding personalized therapeutic strategies.

## Author Contributions

**Fang Guo**: writing–original draft, conceptualization, writing–review and editing, formal analysis, data curation, resources. **Chong Zheng**: writing–original draft, conceptualization, writing–review and editing, formal analysis. **Tao Yang**: conceptualization, writing–review and editing, formal analysis. **Fang‐Wei Hu**: formal analysis, writing–review and editing, conceptualization. **Yan‐Gui Chen**: writing–review and editing, formal analysis, conceptualization. **Chia‐Wei Liou**: writing–review and editing, formal analysis, supervision, project administration. **Bin Cai**: resources, supervision, conceptualization, writing–review and editing, formal analysis, project administration.

## Conflicts of Interest

The authors declare no conflicts of interest.

### Peer Review

The peer review history for this article is available at https://publons.com/publon/10.1002/brb3.70337.

## Data Availability

Research data are not available for sharing.
